# Novel Treatments for Pediatric Relapsed or Refractory Acute B-Cell Lineage Lymphoblastic Leukemia: Precision Medicine Era

**DOI:** 10.3389/fped.2022.923419

**Published:** 2022-06-23

**Authors:** Shang Mengxuan, Zhou Fen, Jin Runming

**Affiliations:** Department of Pediatrics, Union Hospital, Tongji Medical College, Huazhong University of Science and Technology, Wuhan, China

**Keywords:** relapsed or refractory acute lymphoblastic leukemia (R/R ALL), pediatrics, immunotherapy, targeted therapy, personalized therapy

## Abstract

With the markedly increased cure rate for children with newly diagnosed pediatric B-cell acute lymphoblastic leukemia (B-ALL), relapse and refractory B-ALL (R/R B-ALL) remain the primary cause of death worldwide due to the limitations of multidrug chemotherapy. As we now have a more profound understanding of R/R ALL, including the mechanism of recurrence and drug resistance, prognostic indicators, genotypic changes and so on, we can use newly emerging technologies to identify operational molecular targets and find sensitive drugs for individualized treatment. In addition, more promising and innovative immunotherapies and molecular targeted drugs that are expected to kill leukemic cells more effectively while maintaining low toxicity to achieve minimal residual disease (MRD) negativity and better bridge hematopoietic stem cell transplantation (HSCT) have also been widely developed. To date, the prognosis of pediatric patients with R/R B-ALL has been enhanced markedly thanks to the development of novel drugs. This article reviews the new advancements of several promising strategies for pediatric R/R B-ALL.

## Introduction

Acute lymphoblastic leukemia (ALL), particularly B-cell lineage ALL (B-ALL), which has been the most prevalent childhood tumor, is a malignant disease characterized by uncontrolled proliferation of immature lymphoid cells that can invade bone marrow, blood, and extramedullary (EM) sites (1), especially in the central nervous system (CNS) or testicle. Since 1960, with the application of combined chemotherapy, risk stratification, CNS prevention strategies and minimal residual disease (MRD) monitoring, the cure rate of newly diagnosed pediatric ALL has improved steadily. At present, the 5-year overall survival (OS) rate of children with ALL has reached over 90% (2–4); the cumulative recurrence rate has been reduced to less than 10% (5).

Although there have been some significant developments during the past few decades, the outcome of patients with relapsed or refractory (R/R) ALL remains static (6–8). The relapse rate has been reported to be 15–20% in developed countries over the last two decades. However, there has been no standard treatment for patients with R/R ALL, and the long-term survival rate after recurrence is approximately 30∼60% (9–14), depending on the duration of follow-up and the risk groups involved. The 10-year OS and EFS rates were 36 and 30%, respectively, for children treated in the Acute Lymphoblastic Leukemia Relapse Berlin-Frankfurt-Munster (ALL-REZ-BFM) 90 trial (9). Similarly, the 5-year OS and EFS rates of children with first-relapsing ALL treated on the United Kingdom (UK) ALL R2 protocol were 56 and 47%, respectively (15). In Nordic countries, the 5-year OS was 44.7% during 1992–2001 and 57.5% during 2002–2011 (16). With the first relapse of ALL, fewer than 50% of children survive long-term, and the prognosis is even worse for relapses two or later, with a survival rate of approximately 20%.

Of note, R/R ALL is frequently associated with treatment resistance, possibly arising from enrichment of preexisting resistant subclones and/or from mutation acquisition during chemotherapy exposure (2, 17). A comprehensive genomic characterization of the diagnosis-relapse pair observed a dynamic clonal evolution in all cases, with relapse almost exclusively originating from a subclone at diagnosis (18).

Considering that the cause of relapse is related to chemoresistance (19) and that modern chemotherapy regimens have reached the limits of their tolerance, which can no longer improve, it is vital to combine chemotherapy with accurate individualized therapy based on immunotherapy and targeted therapy to amplify the cure rates and quality of life in the future. To date, the continuous development of newly emerging targeted immunotherapies has proven to lead to superior disease-free survival and OS rates, markedly lower toxicities and better MRD clearance. This article focuses on new advancements in several promising strategies for choosing the most proper and effective treatments for R/R B-ALL.

## Overview

### Diagnosis of Relapse and Refractory B-Cell Acute Lymphoblastic Leukemia

Refractory ALL is characterized by poor sensitivity to commonly used chemotherapeutic drugs, a low clinical remission rate and a significantly shortened survival cycle. Schmid et al. (20) stated that refractory acute leukemia can be defined as having at least one of the following conditions: (1) Failure of initial induction therapy after two or more courses of treatment; (2) early recurrence less than 6 months after the first remission; (3) inefficacy to response to induction chemotherapy after recurrence; and (4) multiple relapses.

Relapse of ALL is defined as recurrence of the disease at any site after a period of complete remission, either while still on or after completion of front-line therapy (21). In the BFM group study, very early was defined as less than 18 months from diagnosis; early as more than 18 months from diagnosis and less than 6 months from treatment discontinuation; and late as more than 6 months from treatment discontinuation (22). In the Children’s Oncology Group (COG) study, early diagnosis was less than 36 months from initial diagnosis; late diagnosis was 36 months or more from initial diagnosis. In the St Jude’s Children’s Research Hospital study, early was less than 6 months from completion of frontline therapy; late was 6 months or more from completion of frontline therapy.

### Risk Stratification of Relapse and Refractory B-Cell Acute Lymphoblastic Leukemia

The current treatment protocols for relapsed ALL stratify patients according to their clinical characteristics at diagnosis and relapse and offer different therapeutic options (23).

Previous trials have revealed that the site of relapse (9) and duration of first CR indeed influence both event-free survival (EFS) and OS in childhood ALL (24, 25). In general, isolated bone marrow and early relapse (26) are associated with worse prognoses than isolated extramedullary or late relapse (12, 24, 26).

Nguyen et al. substantiated that the time to relapse, age over 10 years old, presence of CNS disease at diagnosis and male sex were significant predictors of inferior post-relapse (27). Patients suffering very early BM relapse had a particularly terrible outcome, with a survival rate of 0–20% (27, 28), while those experiencing early BM relapses had survival rates from 10 to 40% (29). For late BM relapse patients, survival rates range from 14 to 50% (30).

In addition, persistence of a negative measurable MRD at the end of induction or consolidation therapy correlates with a better chance of surviving after hematopoietic stem cell transplant (HSCT) (31–33), as a number of previous studies have proved that the risk of relapse after transplantation is higher in patients with MRD positivity before HSCT than in those without detectable MRD (34–36).

Moreover, a study conducted by Irving et al. confirmed that TP53 alterations and NR3C1/BTG1 deletions were associated with a higher risk of progression (37). Additionally, DNA methylation, which is a worthy factor, holds prognostic information in relapsed B-ALL (38). Thus, screening relapse patients for key genetic abnormalities will improve prognosis and risk stratification.

## Treatment Strategies

### Multidrug Chemotherapy

Modern R/R B-ALL therapy continues to be based on standard principles, including induction chemotherapy, stem cell transplantation and analogous supportive care (6, 39, 40), particularly the prevention and treatment of infections. Most ALL relapses occur during treatment or within the first 2 years after treatment completion (41). The traditional strategy is to administer chemotherapy drugs at the maximum tolerated dose (MTD), which aims to kill as many tumor cells as possible. However, modern protocols, such as the BFM ALL 2000 protocol, allow for lower treatment dose intensity to improve quality of life, especially for low-risk pediatric patients, while maintaining a high cure rate (42, 43). Moreover, only specific subgroups of patients could rebound from low-intensity chemotherapy (43).

However, some studies have confirmed that low-dose cytarabine and Accra-Adriamycin combined with granulocyte colony-stimulating factor cannot improve the survival prognosis of patients with R/R ALL compared to the Hyper-CVAD regimen (44). A retrospective study evaluated the safety and efficacy of a salvage regimen consisting of G-CSF, low-dose cytarabine, aclarubicin, L-asparaginase and prednisone among R/R ALL patients. The incidence of 2-year OS was approximately 30%, the disease-free survival rate was 15%, and the drug-related mortality rate was 5.6% (45).

Clofarabine, a second-generation purine nucleoside analog, has been approved by the United States Food and Drug Administration (FDA) for treating pediatric R/R ALL patients based on prior phase II studies, with an overall remission rate of 45–55% (46–48). A phase II trial of clofarabine in combination with etoposide and cyclophosphamide conducted in pediatric R/R ALL patients showed encouraging response rates and sustained remission, with an overall remission rate of 44% (39).

Due to multidrug chemotherapy, 70–98% of first-relapse patients achieve a second CR, depending on the risk stratification group (15, 24, 27, 49). Pediatric patients with second or third bone marrow relapse have even poorer outcomes, with only 44 and 27%, respectively, achieving a subsequent CR (50).

**FIGURE 1:**
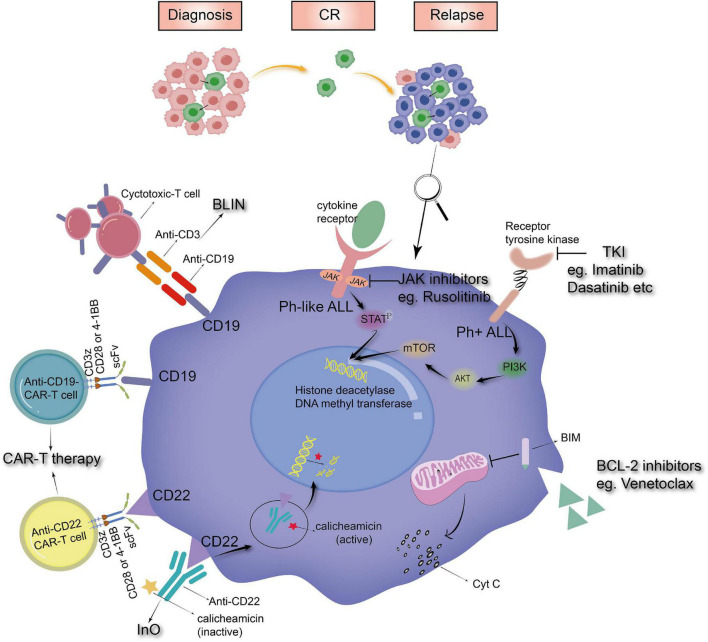
The origin of relapse and the mechanism of novel drugs (BLIN, blinatumomab; InO, inotuzumab ozogamicin; CAR-T therapy, chimeric antigen receptor modified T-cell therapy; TKI, tyrosine kinase inhibitors).

Nevertheless, according to the trials carried out by St. Jude Children’s Hospital, OS rates have been similar recently without a notable upgrade (3, 42, 51), which indicates that the existing treatment has been pushed to its limit. In addition, the prevailing view about the emergence of ALL relapse is that it might originate from a chemoresistant clone (52). These quiescent dormant cells are less sensitive to cytotoxic drugs and hide in niches where the microenvironment confers protection from chemotherapy, escaping therapeutic killing (53–55). Thus, new antileukemic agents are urgently needed. The origin of relapse and the simple mechanism of several promising drugs are displayed in Figure 1 below.

### Immunotherapies

#### Blinatumomab

Blinatumomab, a bispecific monoclonal antibody construct, enables CD3-positive T cells to recognize and eliminate CD19-positive ALL cells (56, 57). It was approved for the treatment of R/R B-cell ALL by the FDA on July 11, 2017 (58). Current studies have demonstrated that blinatumomab has significant monotherapy activity, which induces superior remission among refractory and relapsed patients with markedly lower toxicities in adult patients (59, 60). The main toxicities of blinatumomab are central nervous system injury and cytokine release syndrome (CRS).

Pediatric data using blinatumomab are still quite scarce, and the results of most adult trials and many adult reviews cannot simply be transferred to the pediatric environment due to differences in biology and prognosis between adults and children. According to the existing clinical trials in children, we can obtain such results that regardless of the single-arm test or compared with the current chemotherapy regimen, blinatumomab also shows good efficacy and tolerability. The relevant clinical trials and their results are shown in Table 1.

**TABLE 1:** Clinical trials of blinatumomab in pediatric ALL.

Trial	No. of patients	Efficacy	Toxicities
Multicentric real-life retrospective analyses: in pediatric R/R BCP-ALL (61)	39	CR rate with ≥ 5% blasts: 46% CR rate with ≤ 5% blasts: 81% Median-OS: 16 months Median-relapse-free survival: 33.4 months	Adverse events ≥ grade III: 34.8% Neurotoxicity: 39.1%
A single-center experience: pediatric R/R BCP-ALL (62)	38	Response rate: 34% Complete molecular remission rate: 24%	CRS: 50% Neurotoxicity: 15%
RIALTO trial: in pediatric R/R BCP-ALL (63)	110	Median OS: 14.6 months CR after 2 cycles: 73.5% 1-year OS rate: 87% received allo-HSCT vs. 29% without allo-HSCT	CRS ≥ grade III: 1.5% Neurotoxicity ≥ grade III: 3.6%
Phase III trial: blinatumomab vs. consolidation chemotherapy in pediatric high-risk first-relapse B-ALL (64)	108	MRD remission rate: 90% vs. 54% EFS rate: 69% vs. 43%	Death rate: 14.8% vs. 29.6% Adverse events ≥ grade III: 57.4% vs. 82.4%
Retrospective analyses: in infant ALL with KMT2A-rearranged (65)	11	MRD-negative rate: 100% 3-year EFS rate: 47% 3-year OS rate: 81%	CRS: 27% Neurotoxicity: 9%
Phase III trial: blinatumomab vs. chemotherapy in children and AYAs with high or intermediated risk first-relapse B-ALL (66)	208	2-year OS rate: 71% vs. 58% MRD remission 75% vs. 32%	Infection: 15% vs. 65% Febrile neutropenia: 5% vs. 58% Sepsis: 2% vs. 27% Mucositis: 1% vs. 28%
Phase I/II trial: in pediatric R/R BCP-ALL (67)	70	CR after 2 cycles: 39% MRD-negative rate: 57%	CRS: 11% Neurotoxicity: 24%

*BCP-ALL, B-cell precursor acute lymphoblastic leukemia; R/R ALL, relapsed or refractory acute lymphoblastic leukemia; CR, complete remission; OS, overall survival; EFS, event-free survival; MRD, minimal residual disease; CRS, cytokine release symptom; allo-HSCT, allogeneic hematopoietic stem cell transplant.*

Astonishingly, blinatumomab has particular value in fragile children, such as those with Down Syndrome, or children carrying poor-risk molecular/cytogenetic features (including TCF3-HLF-positive B-ALL) (63, 65), which has greatly encouraged us to apply it to some rare refractory ALL subtypes and as a bridge tool to HSCT.

Instead, patients treated with blinatumomab had fewer side effects of hematological toxicity and infection, whereas central nervous system events, such as encephalopathy, tremor and depression, were more frequent. Clinical trials suggest that the low reactivity of blinatumomab is related to high tumor burden (68). It seems to be a poor choice for patients with bone marrow blasts over 50%, a previous history of leukemia or concurrent extramedullary leukemia.

Of note, to solve the problem of the rapid occurrence of chemoresistance and easy relapse, an innovative treatment without chemotherapy is being studied. To date, in a phase II trial, researchers have treated patients with dasatinib plus glucocorticoids, followed by two cycles of blinatumomab without chemotherapy (69). Of the 63 patients selected, the incidences of CR and OS were 98 and 95%, respectively, with a disease-free survival rate of 88%. This new regimen is feasible, effective and safe, the treatment-related mortality rate is very low, and the overall and disease-free survival outcomes are very promising.

#### Inotuzumab Ozogamicin

Inotuzumab ozogamicin (InO) is an antibody–drug conjugate (ADC) composed of a humanized anti-CD22 monoclonal antibody conjugated with calicheamicin that can induce double-stranded DNA breakage and subsequent cell death. The FDA approved InO for the treatment of R/R B-ALL in February 2017. Presently, *in vitro* experiments have confirmed that the drug is safe and effective against B-line ALL in children, and its main side effect is hepatotoxicity, especially sinus obstruction syndrome after transplantation (70–73).

It was reported that CD22 is expressed in the vast majority of childhood B-cell precursor ALL (BCP-ALL), but pediatric experience with InO is limited. Nevertheless, existing clinical trials have implied that InO can induce deep remission, maximize EFS and OS, and potentially minimize treatment-associated toxicity in patients with R/R ALL, along with a superior CR rate ranging from 45 to 67%, and promote subsequent HSCT or other treatments.

Although most studies have shown that patients receiving InO have better quality of life and superior disease-free survival, hepatotoxicity is more frequent in the InO arm (51% vs. 34%); the incidence of sinusoidal obstruction syndrome (SOS), drug-induced liver injuries (DILI) and venous occlusive disease after InO treatment was 1.5, 7.9, and 11%, respectively, which were seen more frequently than in the control group receiving standardized chemotherapy (1, 1, and 1%) (74). Table 2 lists several relevant InO results applied to pediatrics.

**TABLE 2:** Clinical trials of inotuzumab ozogamicin in pediatric ALL.

Trial	No. of patients	Efficacy	Toxicities
Phase II trial: in pediatric R/R ALL (71)	51	CR rate: 67% MRD-negative rate: 71%	Hepatic transaminitis or hyperbilirubinemia ≥ grade III: 12% Infections ≥ grade III: 22% SOS: 52% after HSCT
Phase II trial: in pediatric R/R ALL (75)	48	CR/CRi rate: 58.3%	ALT elevation ≥ grade III: 6.3% hyperbilirubinemia ≥ grade III: 2.1% SOS ≥ grade III after HSCT: 28.6%
Phase I trial: in pediatric R/R ALL (76)	25	Response rate after DLI: 80% Response rate after DLII: 85% MRD-negative rate: 84% 12-month OS rate: 40%	
Case report: in infants and young children with R/R ALL (72)	15	CR rate: 47% 6-month OS rate: 47%	Veno-occlusive disease: 13% (after HSCT)
Retrospective study: in pediatric BCP-ALL (77)	29	CR rate: 47.6% 12-month OS rate: 45.8% 12-month EFS rate: 27.5%	
French trial: in pediatric CD22-R/R ALL (73)	12	CR/CRi rate: 67% MRD-negative rate: 17% 12-month OS rate: 38% 12-month EFS rate: 33%	Relapse rate: 17% Hepatic toxicities ≥ grade III: 33% Neutropenia ≥ grade III: 83% Thrombocytopenia ≥ grade III: 75% Anemia ≥ grade III: 33% SOS: 50% (after HSCT)

*SOS, sinusoidal obstruction syndrome; DLI, dose level I; DLII, dose level II.*

#### Chimeric Antigen Receptor-T Cells

Chimeric antigen receptor modified T (CAR-T) cell therapy has recently emerged as a promising tactic for treating B-lineage malignancies. It has been developed a great deal recently. The structure of the latest generation of CAR-T cells includes the extracellular antibody domain of a single chain variant fragment (ScFv) targeting the surface markers of leukemic cells, the intracellular T-cell signal domain, including the CD3ζ domain and costimulatory domain, such as 4-1BB or CD28, as well as more functional elements, such as cytokines (78–80). It can be produced not only from autologous T cells but also from allogeneic T cells after a previous allogeneic HSCT at disease recurrence, rarely leading to graft-vs.-host disease. Approval for the treatment of R/R ALL in pediatric and young adult patients has been granted. Additionally, the FDA approved the use of Breyanzi (lisocabtagene maraleucel, liso-cel) for the treatment of R/R large B-cell lymphoma on February 5, 2021 (81). Odora Anagnostou et al. systematically reviewed and analyzed the efficacy of CD19-specific CAR-T-cell therapy, and the CR rate and cumulative recurrence rate after treatment were 81 and 36%, respectively (82). The CR rates of patients with autologous and allogeneic T-cell-derived CAR-T cells were 83 and 34%, respectively. CD22 CAR-T cells are confirmed to be an effective salvage therapy for patients who have experienced relapse after or are refractory to CD19-targeted therapies.

Notably, the control of EM relapse with CAR-T cells and their penetration of the blood–testis barrier and blood–brain barrier have been reported (83–85). In a *post hoc* analysis, 195 patients with relapsed or refractory CD19-positive ALL or lymphocytic lymphoma in which participants received CD19-directed CAR T-cell therapy were recruited. Among these 195 patients, the CR rate was similar between the CNS-positive group and the CNS-negative group (97% vs. 96%), with no significant difference in relapse-free survival (60% vs. 60%) or OS rate (83% vs. 71%). The study confirmed that CAR-T therapy actively clears CNS disease and maintains durable remission in children and young adults with CNS relapsed or refractory B-ALL or lymphocytic lymphoma without increasing the risk of severe neurotoxicity (86).

The most common adverse reactions are CRS (87, 88) and attention deficit or insanity, with incidences of up to 75∼100% (89, 90) and 64% (91), respectively. Other toxicities include infusion reaction, tumor lysis syndrome, allergic reaction and immunogenicity, infection and anti-infection prevention.

With the continuous research and development of CAR-T cells, different modified CAR-T cells have also been developed. The experiments of Magnani et al. suggested that CAR-T cells genetically engineered with sleeping beauty (SB) transposons and differentiated into cytokine-induced killer (CIK) cells have good anti-leukemia activity and no serious side effects (92). Dai et al. conducted a study on CD19/CD22 bispecific CAR T-cell therapy in patients with relapse or inefficacy after CD19 targeted therapy (93). It was confirmed that bispecific CAR T cells could stimulate strong antileukemic activity; the MRD-negative CR rate was 100%, and the incidence of neurotoxicity was 0.

Nevertheless, despite its many advantages over other forms of cancer therapy, including *in vivo* expansion and long-term persistence, treatment with CAR-T cells remains a work in progress. Therefore, the alternative platform of CAR projects and gene editing techniques (such as CRISPR–Cas9 gene editing) have also been introduced into the construction of CARs to overcome the current limitations of this therapy. New alternative platforms include γδ T cells and NK cells. Using γδ T cells from donors as the host of CAR can avoid graft host reaction because these cells lack allogeneic (94, 95). *In vivo* experiments have proven that it is feasible to produce γδ CAR-T effector cells with high purity and high efficiency (96). Another research hotspot is CAR-NK cells, which are cheaper and less toxic than CAR-T cells (97–102). In phase I and phase II trials, 64% of patients with R/R CD19-positive cancers achieved remission after receiving anti-CD19 CAR-NK-cell therapy, with an adverse reaction rate of 0% (103). Similarly, CRISPR/Cas9 technology is unveiling a new era for CAR-T-cell therapy (104–107). Through genome editing technology, it is possible to create the next generation of CAR T-cell products, including universal CAR T cells, by destroying endogenous T-cell receptors (TCRs) or human leukocyte antigens (HLAs), removing inhibitory regulators to produce more powerful CAR T cells, and adding suicide genes or inducible safety switches to create more controllable CAR T cells. The relevant clinical trials and their results are shown in Table 3.

**TABLE 3:** Clinical trials of CAR-T therapy in pediatric ALL.

Trial	No. of patients	Efficacy	Toxicities
Phase II trial: B-ALL in pediatric and young adults (108)	75	CR rate: 81% MRD-negative rate: 100% 6-month OS rate: 90% 6-month EFS rate: 95% 12-month OS rate: 76% 12-month EFS rate: 50%	Adverse events ≥ grade III: 73% CRS: 77% Neurotoxicity: 40%
Phase II trial: EM-ALL in pediatric and young adults (83)	55	CR rate in CNS group: 88% CR rate in non-CNS EM group: 66%	
Multicentric real-life retrospective analyses: CD19-targeted CAR-T in pediatric ALL and non-Hodgkin lymphoma (109)	511	CR rate: 85.5% EFS rate: 52.4% OS rate: 77.2%	CRS ≥ grade III: 11.6% Neurotoxicity ≥ grade III: 7.5%
Phase I trial: CD19-targeted CAR-T in pediatric R/R B-ALL (110)	30	CR/CRi rate: 83.3% MRD-negative rate: 75% 1-year-OS rate: 63% 1-year-EFS rate: 60% 3-year-OS rate: 42% 3-year-EFS rate: 37%	CRS: 90% Neurotoxicity: 15% CRS ≥ grade III: 16.7% Neurotoxicity ≥ grade III: 10%
Phase I trial: in pediatric and young adult Ph-negative R/R B-ALL (111)	52	1-Year-OS rate: 92.3% 1-year-EFS rate: 80.1% 2-year-OS rate: 84.3% 2-year-EFS rate: 76.0%	1-Year relapse rate: 14.1% 2-year relapse rate: 19.7%
A pilot trial: humanized CD19-targeted CAR-T in children and adults with R/R B-ALL and B-lymphoma (112)	74	1-Year relapse-free rate: 84% in CAR-naïve cohort and 74% in retreatment cohort 2-year relapse-free rate: 74% in CAR-naïve cohort and 58% in retreatment cohort	CRS: 84% Neurotoxicity: 39%

### Molecular Targeted Drugs

With the increased understanding of genetic mutations discovered in ALL, treatments targeting genetic mutations and signaling pathways are emerging. As a new landmark therapeutic approach, targeted therapy has been described as a promising treatment for pediatric ALL with specific genetic abnormalities. The main purpose of using molecularly targeted drugs combined with chemotherapy or immunotherapy is to improve outcomes of R/R B-ALL patients (113). Herein, there are many novel molecular targeted drugs under development.

#### Tyrosine Kinase Inhibitors

Of the chromosomal abnormalities described in ALL, the most common is a fusion gene called BCR-ABL. This disorder is classified as Philadelphia chromosome-positive (Ph +) ALL (114–116). Ph + ALL patients account for approximately 3% of pediatric ALL patients. Prior to the emergence of tyrosine kinase inhibitors (TKIs) targeting the BCR-ABL gene, the 5-year EFS of Ph + ALL patients with chemotherapy alone was dismal, at only approximately 30% (117), and the OS rate of chemotherapy combined with HSCT was approximately 45%. However, the incorporation of TKI into the chemotherapy regimen significantly increased the long-term survival rate of Ph + patients, with a long-term survival rate of nearly 60–75% in the imatinib group (118–120) and over 80% in the dasatinib group (119, 121).

The first generation of TKI is imatinib. In patients with recurrent Ph + ALL, the initial CR rate of imatinib is 20–29%, and the CR rate is more than 90% when imatinib is added to intensive chemotherapy (122). The 5-year OS rate and EFS rate of the second-generation TKI dasatinib are 86 and 60%, respectively (123, 124). However, taking into account the rapid development of drug resistance and short response (125–128), a new generation of TKIs, such as ponatinib (129), has been developed. A phase II clinical trial conducted by Cortes et al. (130) on second-generation TKI resistance or intolerance or CML and ALL carrying T315I mutations in adults showed that the 5-year OS and EFS of the patients reached 73 and 53%, respectively, confirming the third-generation BCR-ABL inhibitor ponatinib. It can overcome the problem of drug resistance of second-generation TKIs (131). Its application in adults plays a good role in predicting the application of ponatinib in children with Ph + ALL. The clinical efficacy of ponatinib in children with Ph + ALL needs further study.

#### Janus Kinase Inhibitors

Ph-like ALL has a gene expression pattern similar to Ph + ALL but lacks the BCR-ABL fusion gene (132). The activation of the JAK pathway, which is a cytoplasmic tyrosine kinase with a crucial role in signal transduction from multiple hemopoietic growth-factor (HGF) receptors, is one of the most common abnormal events in Ph-like ALL (133). As the first approved inhibitor of the JAK kinase family, ruxolitinib has particular efficacy against both JAK1 and JAK2 (134) and was approved as the first inhibitor of the JAK family by the FDA and the European Drug Administration (EMA) in 2011 and 2012, respectively (135–138). Another JAK2 inhibitor, pacritinib (139), has also shown exceptional efficacy in hematologic malignancy (140). Other inhibitors, including tofacitinib (141) and peficitinib (142), mainly inhibit JAK3 and have been approved for the treatment of rheumatoid arthritis (RA) in the United States.

Preliminary clinical studies have confirmed that roxolitinib is well tolerated in the treatment of R/R tumors (including leukemia) in children (134, 137). Notably, several Ph + ALL mouse models constructed by Appelmann and Kong et al. confirmed that dasatinib (143) or nilotinib (144) combined with ruxolitinib significantly prolonged the survival time of mice, eliminated leukemia proliferating cells (LPCs) more effectively, and decreased the activity of phosphorylated JAK2 at the molecular level, thus limiting the emergence of ABL1 mutants of dasatinib and reducing drug resistance and recurrence compared to single-agent therapy both *in vitro* and in humanized mice.

In addition, it has been reported that triple intrathecal injection (IT) therapy plus ruxolitinib was well tolerated by Ph-negative B-ALL patients with systemic and central nervous system recurrence and successfully eradicated highly refractory leptomeningeal ALL (132, 145). This case confirms that ruxolitinib combined with chemotherapy can eradicate chemotherapy-resistant CNS leukemia.

#### BCL-2 Inhibitors

The BCL-2 protein family, which is critical for controlling cell survival, has been identified as one of the six hallmarks of cancer (146), containing six anti-apoptotic proteins, such as BCL-2, and several proapoptotic proteins that maintain the balance between cell death and survival. BCL-2, which can confer a survival advantage to tumor cells by preventing apoptosis (147, 148), is particularly dysregulated in multiple human cancers (149–152). Therefore, overexpression of BCL-2 might be associated with chemoresistance. Ventoclax (ABT-199), as an efficient and safe BCL-2 inhibitor, has become a novel method of targeted therapy in ALL. Navitoclax (ABT-263) has been demonstrated as a single agent or in combination with other drugs to successfully ameliorate leukemia progression while easily causing significant thrombocytopenia (153, 154). Other inhibitors include ABT-737 (155).

To date, the unexpected efficacy and potential therapeutic strategies of ventoclax observed in patient-derived xenograft tumor (PDX) models lacking BIM expression and in MLL-rearranged ALL xenografts *in vivo* and *in vitro* have been confirmed (156, 157). Ventoclax combined with other drugs can increase chemosensitivity, prevent drug resistance, and reduce the incidence of dose-dependent side effects of chemotherapy compared to the single agent use of this drug. Moreover, several clinical cases of ETP-ALL have proven that ventoclax combined with low-intensity chemotherapy (158), nelarabine (159), decitabine (160) or bortezomib (161) has a good antileukemia effect.

### Personalized Treatment Based on Novel Techniques

#### Genomic Sequencing

R/R ALL is characterized by clonal heterogeneity. Genetic and epigenetic abnormalities, which can increase the proliferation potential of leukemic cells, drive treatment resistance and eventually give rise to relapse or treatment failures (162). In the last two decades, there have been important genomic discoveries in ALL, such as RNA sequencing (RNA-seq), next-generation sequencing (NGS) and genomic-capture high-throughput sequencing (gc-HTS-seq) (163), which play an essential role in risk stratification and have therapeutic and prognostic implications (163–165).

Notably, comprehensive genomic studies have revolutionized our understanding of the molecular taxonomy of ALL by clarifying the subclassification of ALL. Li et al. performed an initial study to reanalyze and delineate the transcriptome landscape of BCP-ALL through RNA-seq, which identified six additional undescribed gene expression subgroups apart from eight previously described subgroups and revealed related prognostic stratification. In addition, gc-HTS seq also expands the spectrum of suitable MRD targets and allows for the identification of genomic fusions associated with risk and treatment stratification in childhood ALL. Similarly, a novel technique called digital multiplex ligation-dependent probe amplification (digital MLPA™) can provide fast and highly optimized aberration profiling for the decipherment of the clonal origin of relapse and yields extremely relevant information for clinical prognosis assessment (166).

The results described above strongly suggest that genomic techniques should be introduced into the clinical diagnostic workup. Recently, personalized medicine in ALL has been used to characterize patients into prognosis groups based on karyotypes to guide treatment options. The highlights of these techniques include detecting inherited predispositions of ALL, finding relevant molecularly targeted therapies through genomic-defined ALL subtypes and monitoring treatment response *via* pharmacogenomics and novel MRD biomarkers (167). These mutations confer chemoresistance and might have implications for therapeutic decisions. Insights into the genomics of ALL further provide a compelling biologic rationale to expand the scope of precision medicine therapies for childhood ALL. Ultimately, tailored treatment strategies for ALL-related gene damage and pathways might improve antileukemic efficacy, diminish recurrence, and reduce adverse toxicity.

#### High-Throughput Drug Sensitivity Screening

However, the choice of options for follow-up personalized treatment only through abnormities in patients’ genotypes is extremely limited.

Currently, high-throughput drug sensitivity (HDS) screening has already been used for the development of personalized treatment approaches in acute myeloid leukemia (AML). It can improve the long-term survival rate of AML patients through individual molecular characteristics or drug sensitivity profiles (168). A vast number of preliminary clinical trials have exhibited outstanding and encouraging benefits in seeking optimum individualized approaches based on HDS screening (169–171).

Intriguingly, EI Andersson et al. systematically explored the diversity of drug responses in T-cell prolymphocytic leukemia (T-PLL) patient samples by using this platform and correlated the findings with somatic mutations and gene expression profiles. The trial showed that all T-PLL samples were sensitive to SNS-032, a cyclin-dependent kinase inhibitor, indicating previously unexplored targeted tactics for treating T-PLL, which has notoriously chemorefractory behavior (172).

In the absence of targetable mutations, these drug sensitivity screenings can provide treatment options rather than genetic abnormalities. However, HDS screening of anticancer drugs has not yet been generally applied in clinical practice for ALL patients, and it has great potential in personalized treatment, particularly in drug target problems for chemo-resistance groups (173). By performing HDS screening, we can analyze and determine optimal individual dosages combined with or without targeted drugs, which improves treatment efficacy while reducing or avoiding toxicity (174–176). Nevertheless, we still need larger trials to evaluate the utility of these technologies in routine clinical settings along with long turnover times and high costs.

## Conclusion

R/R ALL is still a difficult topic among children. Existing immunotherapy and molecular targeted therapy have proven their effectiveness, feasibility and safety, and they have significantly improved the prognosis of patients since they were approved for use. The latest research results also prove the prognosis of the era without chemotherapy. Moreover, novel techniques, such as whole-genome sequencing, next-generation sequencing and HDS screening, play a vital role in selecting appropriate individualized drugs and reducing treatment failure caused by drug resistance. For the treatment of patients with R/R B-ALL, we need a comprehensive understanding of patients’ genotypes and immune indicators, combined with the course of the disease, economic status and level of medical technology in the country, to choose the appropriate treatment.

In the future, we will further study the molecular mechanism of R/R ALL, design safer and more effective precision medicine to address the chemotherapy resistance and recurrence of tumor cells, prolong life expectancy and enhance the quality of life of pediatric patients. It is believed that the dismal situation of children with R/R ALL will be overcome.

## Author Contributions

SM collected the data and wrote the manuscript. ZF and JR reviewed and finalized the manuscript. All authors have read and agreed to the published version of the manuscript.

## Conflict of Interest

The authors declare that the research was conducted in the absence of any commercial or financial relationships that could be construed as a potential conflict of interest.

## Publisher’s Note

All claims expressed in this article are solely those of the authors and do not necessarily represent those of their affiliated organizations, or those of the publisher, the editors and the reviewers. Any product that may be evaluated in this article, or claim that may be made by its manufacturer, is not guaranteed or endorsed by the publisher.
